# Oncofetal gene SALL4 and prognosis in cancer: A systematic review with meta-analysis

**DOI:** 10.18632/oncotarget.14952

**Published:** 2017-02-01

**Authors:** Lorenzo Nicolè, Tiziana Sanavia, Nicola Veronese, Rocco Cappellesso, Claudio Luchini, Paolo Dabrilli, Ambrogio Fassina

**Affiliations:** ^1^ Department of Medicine, Surgical Pathology & Cytopathology Unit, University of Padova, Padova, Italy; ^2^ Department of Biomedical Informatics, Harvard Medical School, Boston, MA, USA; ^3^ National Research Council, Neuroscience Institute, Aging Branch, Padova, Italy; ^4^ Department of Pathology and Diagnostics, University and Hospital Trust of Verona, Verona, Italy; ^5^ ARC-NET Research Center, University and Hospital Trust of Verona, Verona, Italy; ^6^ Department of Pathology, Santa Chiara Hospital, Trento, Italy; ^7^ Pathological Anatomy and Histology Unit, Veneto Institute of Oncology, Padova, Italy

**Keywords:** SALL4, cancer, prognosis, meta-analysis, targeted therapy

## Abstract

The Spalt-Like Transcription Factor 4 (SALL4) oncogene plays a central function in embryo-fetal development and is absent in differentiated tissues. Evidence suggests that it can be reactivated in several cancers worsening the prognosis. We aimed at investigating the risk associated with SALL4 reactivation for all-cause mortality and recurrence in cancer using the current literature. A PubMed and SCOPUS search until 1st September 2016 was performed, focusing on perspective studies reporting prognostic parameters in cancer data. In addition, 17 datasets of different cancer types from The Cancer Genome Atlas were considered. A total of 9,947 participants across 40 cohorts, followed-up for about 5 years on average, were analyzed comparing patients showing SALL4 presence (SALL4+, n = 1,811) or absence (SALL4-, n = 8,136). All data were summarised using risk ratios (RRs) for the number of deaths/recurrences and hazard ratios (HRs) for the time-dependent risk related to SALL4+, adjusted for potential confounders. SALL4+ significantly increased overall mortality (RR = 1.34, 95% confidence intervals (CI)=1.21-1.48, p<0.0001, I^2^=66%; HR=1.4; 95%CI: 1.19-1.65; p<0.0001; I^2^=63%) and recurrence of disease (RR = 1.25, 95% CI = 1.1-1.42, p=0.0006, I^2^=62%); HR=1.52; 95% CI: 1.22-1.89, p=0.0002; I^2^=69%) compared to SALL4-. Moreover, SALL4 remained significantly associated with poor prognosis even using HRs adjusted for potential confounders (overall mortality: HR=1.4; 95%CI: 1.19-1.65; p<0.0001; I^2^=63%; recurrence of disease: HR=1.52; 95% CI: 1.22-1.89, p=0.0002; I^2^=69%). These results suggest that SALL4 expression increases both mortality and recurrence of cancer, confirming this gene as an important prognostic marker and a potential target for personalized medicine.

## INTRODUCTION

The stem-like phenotype in cancer is the result of epigenetic and genetic alterations leading to the expression of genes involved in cell migration, invasion, angiogenesis, self-renewal, anti-apoptosis, and immune-escape, which are fundamental for the embryo-fetal development. The expression of a stem-like phenotype seems to play a central role in defining the malignant potential of different cancers. During the last decades, several stemness-related genes have been proposed as diagnostic markers for cancer, sometimes with prognostic significance. In particular, the fetal oncogene *Spalt-Like Transcription Factor 4* (*SALL4*) has recently emerged as a potential prognostic marker in many tumors. *SALL4* encodes for a zinc-finger transcription factor that plays an essential role during embryo-fetal development forming a regulatory network with other stemness-related genes, such as the *Octamer-Binding Transcription Factor 4* (*OCT-4*), the *Nanog Homeobox* (*NANOG*), and the *Sex-Determining Region Y-Box 2* (*SOX2*), [1–3] and then gradually disappears until it remains strongly silenced in fully differentiated tissues (except for the germline cells and the hematopoietic stem/progenitor cells) [4, 5].

Analyses of *SALL4* expression and its epigenetic status as well as studies on cellular models have shown its oncogenic role in several tumors, such as precursor B-cell lymphoblastic lymphoma, acute and chronic myeloid leukemia, gastrointestinal, breast, and lung cancers. *SALL4* expression is generally assessed by immunohistochemistry (IHC) on whole section or tissue microarray (TMA), or by molecular testing, such as real time PCR and methylation analysis of the promoter region. Recently, several studies have proposed SALL4 as possible prognostic marker for cancer [6]. However, due to the lack of a comprehensive investigation of its prognostic value and to the different assessment techniques and protocols, the reliability of SALL4 as prognostic marker in cancer is still doubtful. In this work, we presented a systematic review and meta-analysis in order to investigate the prognostic role of SALL4 presence (SALL4+) in cancer patients by considering all-cause mortality and recurrence of cancer, evaluating whether SALL4+ can be associated with a poorer prognosis with respect to the absence of SALL4 (SALL4-).

## RESULTS

### Study and patient characteristics

The bibliographic search included 175 not-redundant articles. After excluding 137 articles based on title/abstract review, 38 articles were retrieved for full text review. 22 studies published after 2012 [7–29] were collected, reporting 23 independent cohorts (Supplementary Figure 1). The quality of the studies, assessed through NOS score [30], was generally good without any study with possible high risk of bias (Supplementary Table 1). Most of these 22 studies were carried out in Asia (n=18), three in USA, and one in Europe, mainly focusing on hepatocellular carcinoma (HCC) (n=11) and SALL4 was mainly assayed by IHC (n=18) rather than by molecular tests (n=6). In addition, 17 datasets of The Cancer Genome Atlas (TCGA) [31] were selected (see Methods section for additional details). All these studies were carried out in USA and SALL4 transcriptional activity was investigated by RNA sequencing technology. The selected TCGA datasets represent the following cancer types: Bladder Urothelial Carcinoma (BLCA), Breast invasive carcinoma (BRCA), Cervical squamous cell carcinoma and endocervical adenocarcinoma (CESC), Colon and Rectum adenocarcinoma (COADREAD), Esophageal carcinoma (ESCA), Glioblastoma multiforme (GBM), Head and Neck squamous cell carcinoma (HNSC), Kidney renal clear cell carcinoma (KIRC), Liver hepatocellular carcinoma (LIHC), Lung adenocarcinoma (LUAD), Lung squamous cell carcinoma (LUSC), Ovarian serous cystadenocarcinoma (OV), Sarcoma (SARC), Skin Cutaneous Melanoma (SKCM), Stomach adenocarcinoma (STAD), Thyroid carcinoma (THCA) and Uterine Corpus Endometrial Carcinoma (UCEC).

40 different cohorts were considered for the meta-analysis, screening 9,947 patients (1,811 SALL4+ vs. 8,136 SALL4-) with a median follow-up period of 63.75 months (range: 7-250).

After dividing the participants by SALL4 status, no significant differences were found in terms of age (p=0.54), gender (p=0.9), tumor node metastasis (TNM) stage I-II percentage (p=0.21), grade (p=0.22), nodal (p=0.4) or vascular (p=0.31) invasion. Full descriptive details of all data are reported in Supplementary Tables 2a-2b.

### Risk ratios and adjusted hazard ratios for overall and disease-free survival

The presence of SALL4 resulted significantly associated with higher death rate (55.2% SALL4+ vs. 39.8% SALL4- on average, RR=1.34; 95%CI: 1.21-1.48, p<0.0001; I^2^=66%) (Figure 1a). Similar findings were obtained also considering the recurrence of cancer as outcome (26 cohorts [12, 14, 16, 17, 23, 25, 28 and the TCGA cohorts]; recurrence rate: 51.5% SALL4+ vs. 42.2% SALL4-; RR=1.25; 95% CI: 1.1-1.42, p=0.0006; I^2^=62%) (Figure 1b). We then investigated the time-dependent risk of death/recurrence in terms of hazard ratios (adjusted for the maximum number of the covariates available in each study). 25 cohorts [14, 16, 18, 19, 23, 25, 26, 28 and the 17 TCGA cohorts] reported a survival analysis adjusted for a number of covariates ranging from 1 to 14 (Supplementary Table 3).

**Figure 1A:**
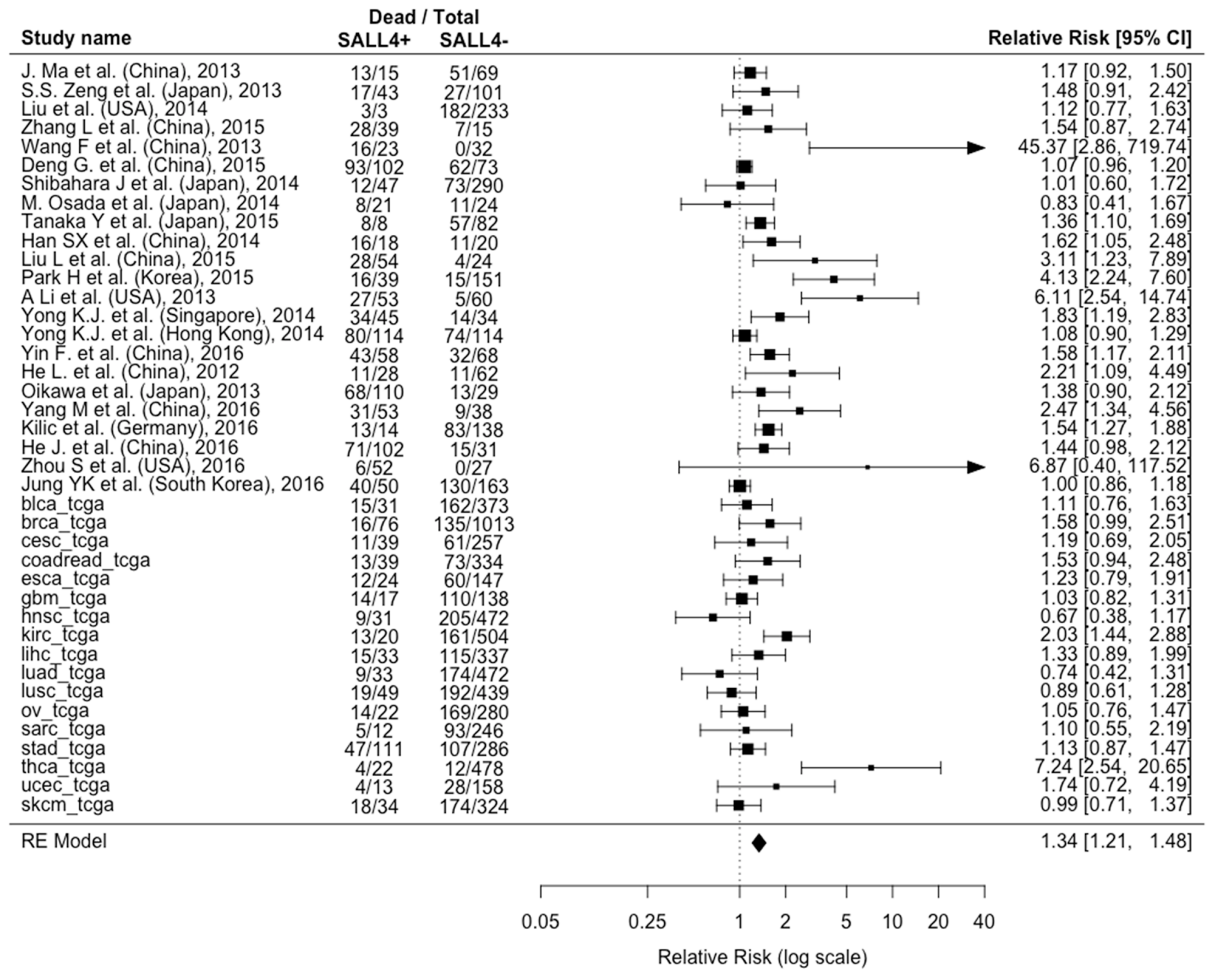
Forest plot of the association between associating the presence/absence of SALL4 with and all-cause mortality Abbreviations: CI: Confidence Interval, SALL4: Spalt-Like Transcription Factor 4, RE: Random-Effects.

**Figure 1B:**
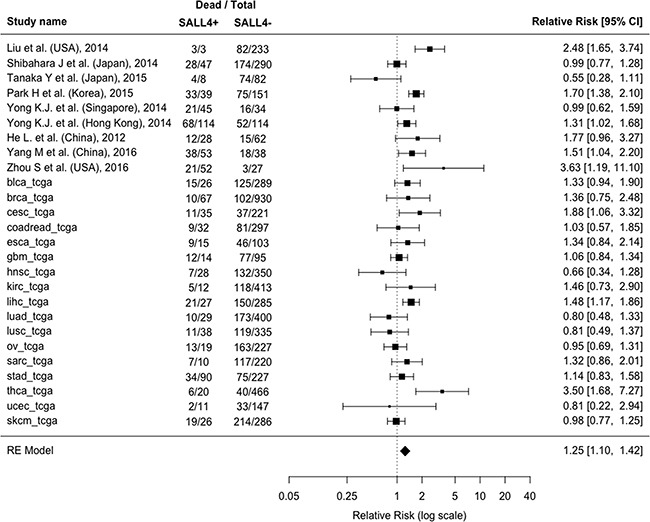
Forest plot of the association between associating the presence/absence of SALL4 with recurrence of cancer Abbreviations: CI: Confidence Interval, SALL4: Spalt-Like Transcription Factor 4, RE: Random-Effects.

About all-causes mortality, the pooled adjusted HR showed a risk of death in SALL4+ patients significantly higher compared to SALL4- patients (HR=1.4; 95% CI: 1.19-1.65; p<0.0001; I^2^=63%) (Figure 2a). For the cancer recurrence, considering the available 4 studies [16, 23, 25, 28] and the 17 TCGA cohorts, a similar result was obtained confirming a worse prognostic role of SALL4+ (HR=1.52; 95% CI: 1.22-1.89, p=0.0002; I^2^=69%) (Figure 2b).

**Figure 2A:**
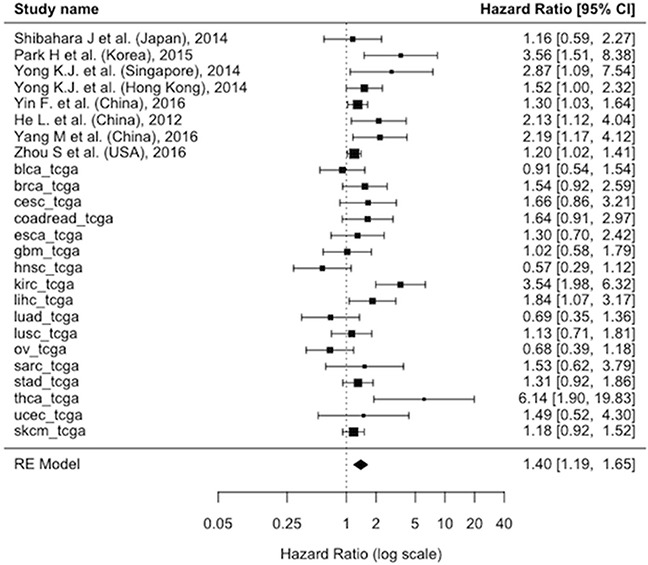
Forest plot of the association associating the presence/absence of between SALL4 with and all-cause mortality using the hazard ratios adjusted for the maximum number of potential confounders available in each paper Abbreviations: CI: Confidence Interval, SALL4: Spalt-Like Transcription Factor 4, RE: Random-Effects.

**Figure 2B:**
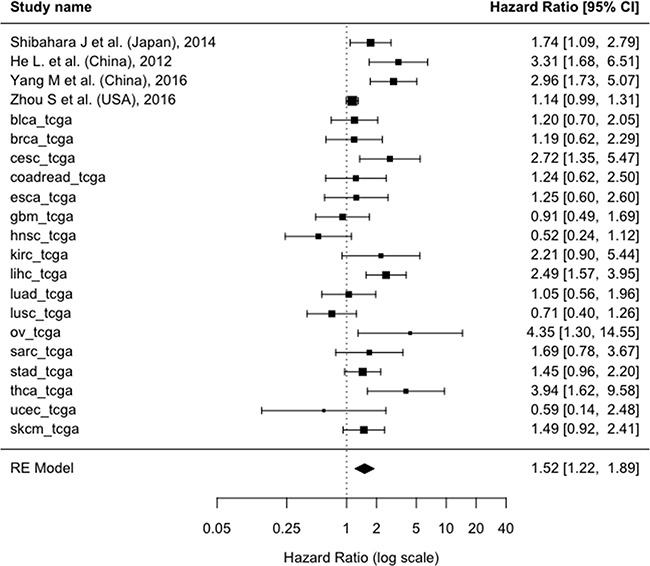
Forest plot of the association associating the presence/absence of between SALL4 with recurrence of cancer using the hazard ratios adjusted for the maximum number of potential confounders available in each paper Abbreviations: CI: Confidence Interval, SALL4: Spalt-Like Transcription Factor 4, RE: Random-Effects.

### Meta-regression, sensitivity analyses and publication bias

The outcomes showed high heterogeneity (as I^2^≥50%, p<0.001), therefore the effects of possible moderators were also considered in the meta-analysis models in order to check whether they can explain to some extent the observed heterogeneity.

In Table 1, the sensitivity analysis for pre-planned moderators (i.e. continent, type of cancer, method of assessment of SALL4) seems to poorly explain this heterogeneity. It is worth to stress the paucity of the studies for some strata (e.g. for the hazard ratio of cancer recurrence), which does not allow a robust assessment for the effect of some moderators. Table 2 reports the meta-regression analyses for other potential moderators: difference of mean age in SALL4+ vs. SALL4- patients, and differences of their percentages in terms of female number, low stage/high grade tumors, node metastases, vascular invasions. Follow-up period and number of adjustments in the survival analysis were considered as well. The results showed that a possible moderator for both relative risk and adjusted hazard ratio in all-cause mortality can be tumor stage (p<0.05). However, the residual heterogeneity test demonstrated that there should be also other moderators, not available for the observed data, able to influence the overall survival (p<0.005).

**Table 1:** Stratification for some potential categorical moderator variables describing the association between SALL4 and all-cause mortality or recurrence of cancer

Moderator	Strata	Analysis details	RR for all-cause mortality	RR for recurrence of cancer	HR for all-cause mortality	HR for recurrence of cancer
**Continent**	**Asia**	***Pooled estimate***, RR/HR (95%CI) P-value for RR/HR ***Heterogeneity***, *I^2^* (P-value)	1.42 (1.24-1.63) <0.0001 70 (<0.0001)	1.25 (0.98-1.58) 0.07 69 (0.004)	1.71 (1.3-2.24) <0.0001 41 (0.12)	2.46 (1.64-3.7) <0.0001 38 (0.2)
	**Others**	***Number of studiesPooled estimate***, RR/HR (95%CI) P-value for RR/HR ***Heterogeneity***, *I^2^* (P-value) ***Number of studies*** P-value *	20 1.26 (1.06-1.48) 0.0075 63 (<0.0001) 20 0.19	7 1.25 (1.07-1.47) 0.005 60 (0.0004) 19 0.96	7 1.28 (1.07-1.53) 0.007 57 (0.002) 18 0.08	3 1.37 (1.11-1.68) 0.003 57 (0.001) 18 0.02
**Type of cancer**	**HCC**	***Pooled estimate***, RR/HR (95%CI) P-value for RR/HR ***Heterogeneity***, *I^2^* (P-value)	1.35 (1.14-1.61) 0.000569 (0.0004)	1.32 (1.03-1.69) 0.03 78 (0.0001)	1.61 (1.23-2.11) 0.0006 39 (0.15)	2.09 (1.47-2.97) <0.0001 12 (0.29)
	**Others**	***Number of studies*** ***Pooled estimate***, RR/HR (95%CI) P-value for RR/HR ***Heterogeneity***, *I^2^* (P-value) ***Number of studies*** P-value^*^	11 1.34 (1.17-1.53) <0.0001 67 (<0.0001) 29 0.85	7 1.2 (1.04-1.39) 0.01 44 (0.02) 19 0.51	6 1.32 (1.1-1.58) 0.003 58 (0.0008) 19 0.21	2 1.45 (1.16-1.8) 0.001 63 (0.001) 19 0.24
**SALL4 assessment**	**IHC**	***Pooled estimate***, RR/HR (95%CI) P-value for RR/HR ***Heterogeneity***, *I^2^* (P-value)	1.5 (1.26-1.78) <0.0001 77 (<0.0001)	1.5 (1.18-1.89) 0.0008 70 (0.001)	1.54 (1.24-1.92) 0.0001 50 (0.05)	1.99 (1.14-3.47) 0.02 86 (<0.0001)
	**Others**	***Number of studies*** ***Pooled estimate***, RR/HR (95%CI) P-value for RR/HR ***Heterogeneity***, *I^2^* (P-value) ***Number of studies*** P-value ^*^	18 1.24 (1.09-1.4) 0.0008 52 (0.003) 22 0.12	8 1.14 (0.99-1.32) 0.06 49 (0.01) 18 0.05	8 1.29 (1.05-1.6) 0.02 59 (0.001) 17 0.19	4 1.4 (1.1-1.79) 0.006 56 (0.003) 17 0.24

*P-value represents the interaction between strata.

Abbreviations: CI: confidence interval, RR: Relative Risk, HCC: hepatocellular carcinoma, SALL4: Spalt-Like Transcription Factor 4, IHC: immunohistochemistry.

**Table 2:** Meta-regression analysis of potential continuous moderator variables the association between SALL4 and all-cause mortality or recurrence of cancer

	RR for all-cause mortality	RR for Recurrence of cancer	HR for all-cause mortality	HR for Recurrence of cancer
**Difference in mean age**	−0.003 (−0.02 to 0.01)(23 studies, p=0.73)	−0.01 (−0.04 to 0.02)(19 studies, p=0.63)	−0.02 (−0.08 to 0.04)(18 studies, p=0.49)	−0.003 (−0.06 to 0.06)(18 studies, p=0.91)
**Difference in women (%)**	−0.001 (−0.01 to 0.01)(36 studies, p=0.77)	−0.003 (−0.01 to 0.01)(26 studies, p=0.5)	0.004 (−0.01 to 0.02)(25 studies, p=0.59)	0.01 (−0.01 to 0.03)(21 studies, p=0.33)
**Difference in TNM 1-2 (%)**	**−0.01 (−0.02 to 0.001)(30 studies, p=0.03)**	−0.005 (−0.01 to 0.004)(19 studies, p=0.3)	**−0.02 (−0.03 to -0.003)(20 studies, p=0.01)**	−0.01 (−0.03 to 0.01)(16 studies, p=0.21)
**Difference in G3 (%)**	−0.002 (−0.01 to 0.01)(21 studies, p=0.68)	0.01 (−0.002 to 0.01)(13 studies, p=0.16)	0.01 (−0.002 to -0.03)(13 studies, p=0.1)	0.01 (−0.01 to 0.03)(11 studies, p=0.35)
**Difference in nodes metastasis (%)**	0.001 (−0.02 to 0.02)(7 studies, p=0.96)	Few studies	Few studies	Few studies
**Difference in vascular invasion (%)**	−0.002 (−0.01 to 0.01)(10 studies, p=0.73)	0.001 (−0.03 to 0.03)(5 studies, p=0.93)	−0.01 (−0.04 to 0.02)(5 studies, p=0.65)	Few studies
**Follow-up duration (months)**	0.002 (−4e05 to 0.004)(39 studies, p=0.05)	−0.0001 (−0.002 to 0.002)(24 studies, p=0.93)	−0.003 (−0.0001 to 0.01)(25 studies, p=0.06)	0.0002 (−0.004 to 0.01)(21 studies, p=0.5)
**Number of adjustments**	Not applied	Not applied	0.02 (−0.04 to 0.07)(25 studies, p=0.56)	0.03 (−0.03 to 0.1)(21 studies, p=0.34)

All differences are calculated as values in SALL4+ vs. SALL4-, except follow-up duration and number of adjustments.

The data in cells are expressed as coefficients of the meta-regression model (with 95% confidence intervals).

Bold: significant results (p<0.05)

Abbreviations: SALL4: Spalt-Like Transcription Factor 4, RR: relative risk, HR: hazard ratio. TNM: Tumor Staging system, G: tumor grading.

**Figure 3:**
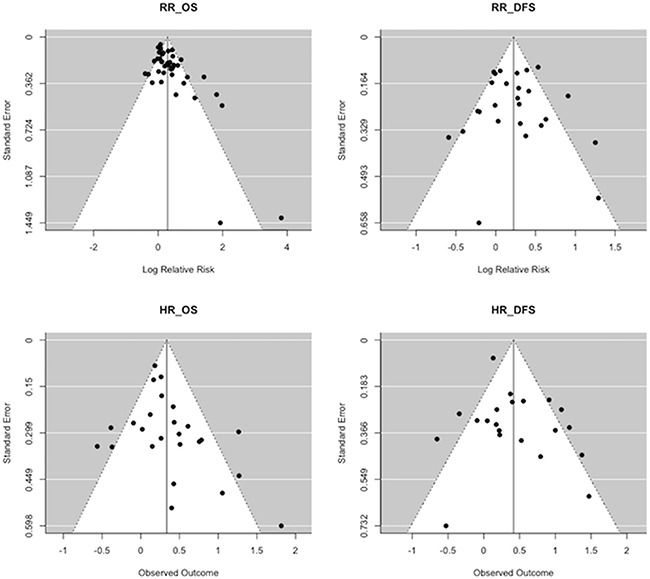
Funnel plots indicating that the publication bias was unlikely across all the outcomes, except for the relative risk in all-cause mortality

Funnel plots inspection (Figure 3) and Egger's test results (Table 3) showed that the publication bias was unlikely across all the outcomes, except for the relative risk in all-cause mortality. However, the trim-and-fill analysis suggested that there is a low risk of publication bias. Moreover, the high fail-safe number for each outcome confirmed the significance of our findings.

**Table 3:** Publication bias analysis

Outcome	Publication bias			Classic fail safe N
	Egger bias (z-score)p value	Kendall's tau>p-value	Trim and fill (95% CI)[trimmed]	
RR for all cause mortality	**4.15p < 0.0001**	**0.31p=0.004**	**1.22 (1.09-1.37)**[8]	**1278**
RR for recurrence of cancer	0.52p=0.6	0.02p=0.9	1.25 (1.1-1.42)[0]	279
HR for all cause mortality	1.98p=0.05	0.23p=0.1	1.2 (0.98-1.46)[6]	361
HR for recurrence of cancer	0.56p=0.57	0.070.7	1.52 (1.22-1.89)[0]	285

Bold: significant results (p<0.05)

**Abbreviations:** RR: relative risk, HR: hazard ratio, CI: confidence interval.

## DISCUSSION

*SALL4* gene exerts its physiological role during embryo-fetal development, then gradually disappears and remains silenced in fully differentiated tissue, with the exception of germ cells and the hematopoietic stem/progenitor cells [4, 5]. Homozygous loss-of function mutation of *SALL4* are lethal for the embryo, while heterozygous mutations cause renal-ocular syndrome and the Okihiro syndrome, associated with multi limbs defects, deficient eye movements, renal malformations, and deafness [32]. As for OCT4, evidence suggests that SALL4 is a major stemness factor and both SALL4 and OCT4 are expressed from the 2-cells stage embryo [33]. Moreover, SALL4 and OCT4 form a transcriptional auto-regulating core network, interacting with NANOG and SOX2 to form a multi-protein complex able to directly regulate both their own expression and the expression of hundreds of downstream target genes involved in pluripotency maintenance, such as *Estrogen Related Receptor Beta* (*ESSRB*)*, REST Corepressor 2* (*RCOR2*)*, Replication Timing Regulatory Factor 1* (*RIF1*) [34]. In addition, SALL4 complex can control gene accessibility recruiting the epigenetic repressor complex Mi-2/Nucleosome Remodelling and Deacetylase (NuRD) involved in *Phosphatase and TENsin homologue* (*PTEN*) and other SALL-family genes down-regulation [35]. The regulation of SALL4 expression is largely unknown. Putative upstream regulators of *SALL4* include several promoter activators such as the Signal Transducer and Activator of Transcription 3 (STAT3), the Wnt/β-catenin pathway, and the Caudal type homeobox 1 (CDX1) [6]. Epigenetic regulation of SALL4 has also been proposed, but it needs further confirmation. Specifically, in induced pluripotent stem (iPS) cells, in embryonal stem cells (ESCs), and in SALL4+ cancers, the expression of SALL4 has been related to the hypo-methylation of its promoter [36]. Moreover, post-transcriptional regulation of SALL4 has been reported and, in particular, the inverse relationship between miR-107 and its expression on human glioma [37].

In cancers where aberrantly reactivated, SALL4 has been associated with the expression of many stemness-related genes (i.e. *OCT4*, *NANOG*, *c-Myc*, and *SOX2*) conferring to the cancer cells self-renewal pluripotency abilities [3]. Moreover, SALL4 expression allows cancer cells to acquire a stem-like phenotype, including: 1) increased mobility, invasion, and neoangiogenetic functions through the expression of epithelial-to-mesenchymal transition-related genes (*e.g. SNAI1, CXCR4, TWIST1, CDH1, Vimentin*, and *ZEB1*) [38]; 2) silencing of pro-apoptotic genes (*e.g PTEN*); 3) inducing the expression of chemo-resistance-related genes (*e.g*. *ATP-Binding Cassette Multidrug Transporter*) [39]; 4) acquisition of immune evasion abilities. All the above-mentioned features are related with the biological behavior of the SALL4+ cancers, which are more aggressive and associated with a worse prognosis compared with SALL4- ones. Recently, a meta-analysis showed a possible correlation between SALL4 expression and poor prognosis in cancer patients [40]. However, a large systematic evaluation about the role of SALL4 expression as prognostic marker including also the large amount of data available from public databases is still lacking. In this study, 22 perspective studies retrieved by the literature and 17 different TCGA cohorts were considered for the meta-analysis, involving 9,947 participants during a median follow-up period of about 5 years. Results showed that the expression of SALL4 was significantly associated with increased cancer mortality and recurrence, also after adjusting for potential confounders in the survival analyses. These findings suggest that the expression of this gene should be early assessed in patients. Therefore, the impact of SALL4 expression analysis should not be considered as a diagnostic and/or prognostic tool only, but it should be also investigated as possible target for new personalized treatments.

As a HCC prognostic factor, SALL4 seems to be particularly useful, as suggested by most of these 22 studies (11 HCC focused) and by the large TCGA dataset of HCC included in our analysis. However, these studies were mostly performed on patients with resectable tumors, thus with early stage of disease, for which the survival is longer as demonstrated by the mean follow-up period of 5 years; and so far a large investigation of SALL4 expression in advanced HCC is still lacking.

Interestingly, although SALL4 was discovered in 1994 [41, 42], all the studies included in this study were published only after 2012, highlighting that its role as prognostic marker and possible molecular target has been investigated only recently. SALL4 was firstly studied in germline cells from solid tumors and it resulted a valid diagnostic marker with a good sensitivity [43], but only later it was associated with poor prognosis in digestive system cancers [18]. A main point of our study was the long follow-up period (5 years on average), which was proper to evaluate the outcomes.

From a methodological point of view, most of the perspective studies used IHC, the most affordable technique to test indirectly genes expression, especially for large case studies. An advantage of IHC is the possibility to evaluate the expression of a marker in the whole tumor section allowing the detection of even a focal positivity in heterogeneous tumors (as it could be for SALL4, a stemness-related marker). However, two studies applied IHC on tissue microarrays instead of whole tumor sections and the low number of tissue cores obtained from each tumor sample could represent a limitation, increasing the risk of false negative cases [43]. In addition, some inconsistencies emerged by comparing the results from IHC studies, probably related to the different antibodies, IHC protocols and assessment criteria. Consensus standardization on SALL4 IHC would improve the reproducibility of the results. Real time PCR (RT-PCR) allows a precise quantification of SALL4 expression, although this method can be affected by tumor heterogeneity and it does not provide any information about the subcellular localization of SALL4. Looking at the TCGA datasets, SALL4 activity was investigated by RNA sequencing, which potentially allows the study of its expression in the entire transcriptome landscape, revealing possible regulative networks of SALL4 and related genes. However, as underlined for RT-PCR, gene expression could be affected by tumor heterogeneity, especially if the sample is not representative of the entire tumor mass. IHC, instead, can differently localize nuclear and cytoplasmic regions, allowing the detection of the position where a transcription factor is supposed to be active. Therefore, in our opinion, the best method to assess SALL4 presence in cancer is standardized IHC on whole tumor section.

The findings of our meta-analysis should be interpreted within their limitations and the most important of them is the inclusion of a limited number of studies. Secondly, through sensitivity and meta-regression analyses we were not able not explain the high heterogeneity found for the investigated outcomes. Finally, in the adjusted survival analyses, several important confounders (like cardiovascular diseases, disability, or other co-morbidities) were not available, further limiting the results. Despite these limitations, our meta-analysis suggests that SALL4 expression shortens overall survival as well as increases the rate of recurrences, even taking in consideration potential confounders. Since many epithelial cancers are characterized by SALL4 reactivation, this gene should be considered for developing future targeted therapeutic strategies. Due to its prognostic value, SALL4 expression should be considered as potential marker in the next-generation histopathological diagnosis, hopefully by standardized IHC protocols, integrating cancer morphological features and molecular targets information [44–52].

## MATERIALS AND METHODS

### Literature search strategy

Two investigators independently conducted a literature search using PubMed and SCOPUS with no language restriction, from database inception to 1^st^ September 2016, for perspective studies comparing relevant outcomes (i.e., all-cause mortality, cancer mortality and recurrence of disease/cancer) in patients with a diagnosis of cancer with loss vs. presence of expression of SALL4. In PubMed, the following search strategy was used: (“SALL4” OR “NM_020436”) AND (mortality OR mortalities OR fatality OR fatalities OR death* OR survival OR prognosis OR “hazard ratio” OR HR” OR “relative risk” OR “RR” OR “prognosis” OR “progression” OR “disease free survival” OR “DFS”). Conference abstracts and reference lists of included articles and those relevant to the topic were also hand-searched for identification of additional relevant articles. Any inconsistencies were resolved by consensus.

### Study selection and quality assessment

Newcastle-Ottawa Scale (NOS) was used to evaluate study quality [30]. This systematic review was performed following the Meta-Analysis Of Observational Studies in Epidemiology (MOOSE) [53] guidelines and Preferred Reporting Items for Systematic reviews and Meta-Analyses (PRISMA) [54] statement. Inclusion criteria for this meta-analysis were: 1) perspective, longitudinal cohort study, 2) immuno-histochemical or molecular investigation of SALL4 expression, 3) diagnosis of cancer, 4) data about mortality or cancer recurrence. Exclusion criteria were: 1) no presence of cancer, 2) no data about relevant outcomes in the title/abstract, 3) no comparison between patients with SALL4+ vs. SALL4-, and 4) *in vitro* or animal research. To avoid overlaps between cohorts, in the presence of two or more studies from the same patient cohort, only the more recent study was taken into account and included in the meta-analysis.

### Data extraction

Two investigators extracted key data from the included articles and a third independent investigator checked these data. For each study, information about authors, publication year, continent, histotype, SALL4 assessment methods, other genes analyzed, participant characteristics according by SALL4 expression data (e.g., age, gender, tumor stage and grading, percentage of participants with nodal metastasis and vascular invasion), number and type of adjustments in survival analysis, and the period of follow-up. When some information was missing, first and/or corresponding authors of the original article were contacted at least four times to obtain unpublished data.

In addition to these studies, one investigator retrieved molecular and clinical data of different cancer types from cBioPortal for Cancer Genomics (http://www.cbioportal.org) using the R package cgdsr version 1.2.5 (http://cran.r-project.org/web/packages/cgdsr/index.html). This package provides R functions for querying the Cancer Genomic Data Server (CGDS) hosted by the Computational Biology Center (cBio) at the Memorial Sloan-Kettering Cancer Center. For each dataset, SALL4 expression levels from RNAseq data were downloaded as z-scores, representing the number of standard deviations from the mean of expression using tumors diploid as the reference population. Datasets reporting a low number of subjects per class for the survival analysis (i.e. less than 10 individuals) were not considered.

### Outcomes

The primary outcomes were number of deaths independent of the cause (all-cause mortality) and number of cancer recurrences during the follow-up period, in relation with the SALL4 presence or absence. The number of deaths due to cancer was preliminary considered as primary outcome, but no perspective study reported this information. As secondary outcomes, we considered hazard ratios (HRs) for all-cause mortality and recurrence, adjusted for the maximum number of confounders present for each study. Survival analysis was performed on TCGA data using the R packages survival version 2.39-5 (https://cran.r-project.org/web/packages/survival/index.html). SALL4 expression was classified in a binary way: one class of patients was identified by the presence of SALL4 (SALL4+) according to an over-expression quantified by a z-score>=2 and the other class was characterized by samples reporting a z-score<2 (SALL4-). For each TCGA dataset, HR for all-cause mortality and recurrence was estimated. Possible confounders of the final model were selected with a step-down procedure: the decision to remove confounders was based on a chi-square test for goodness of fit. All the available confounders were first included in the full model considering main effects only, and then they were sequentially removed if their removal did not result in a significant change of the estimates, using a threshold of 0.05 on the resulting p-values.

### Data synthesis and statistical analysis

All statistical analyses were performed using R language version 3.3.0. R package metaphor version 1.9-9 (https://cran.r-project.org/web/packages/metafor/index.html) was used for the meta-analysis.

Descriptive characteristics of the patients, divided according to the presence or absence of SALL4, were compared using Wilcoxon rank-sum test. In particular, gender, tumor stage and tumor grading were represented in terms of percentages of females, patients with low-stage tumors (i.e. I-II) and patients with high-grade tumors (i.e. G3, G4), respectively. Vascular invasion and node metastasis were represented as percentages as well.

Pooled risk ratios (RRs) and adjusted hazard ratios (HRs) with 95% CIs were calculated for all-cause mortality and recurrence of cancer in patients with SALL4+ vs. SALL4- patients using DerSimonian-Laird random-effects models [55]. Heterogeneity across studies was assessed by the Cochrane I^2^ metric and chi square statistics. Given significant heterogeneity (p<0.05), meta-regression and sensitivity analyses were performed considering potential moderators and according to SALL4 status [56]. The following moderators were tested independently: continent (categorized as Asia vs. other continents), type of cancer (HCC or others), assessment methods (IHC or others), period of follow-up, number of adjustments applied in the survival model, and differences between SALL4+ and SALL4- participants in mean age, gender, tumor stage (divided in TNM stage 1-2, indicating low stage in percentage of SALL4+ vs. SALL4-patients), tumor grading (G3/G4, indicating high grade in percentage of SALL4+ vs. SALL4 patients), node metastasis and vascular invasions as percentage in SALL4+ vs. SALL4- patients.

Publication bias was assessed by visually inspecting funnel plots and using Egger's bias test [56] and Begg-Mazumdar Kendall tau [57]. Then, to account for publication bias, we used the trim-and-fill method, based on the assumption that the effect sizes of all the studies are normally distributed around the center of a funnel plot; in the event of asymmetries, it adjusts for the potential effect of unpublished (imputed) studies [58].

For all the analyses, a p-value less than 0.05 was considered statistically significant.

## SUPPLEMENTARY FIGURE AND TABLES




