# Supplementary material for: Oncofetal gene SALL4 and prognosis in cancer: A systematic review with meta-analysis
Source: Oncotarget. 2017 Feb 1;8(14):22968–79. doi: 10.18632/oncotarget.14952 (PMC5410278; doi:10.18632/oncotarget.14952)
Supplement: Supplementary file 1 [file oncotarget-08-22968-s001.pdf]

Oncofetal gene SALL4 and prognosis in cancer: A systematic review with meta-analysis

SUPPLEMENTARY FIGURE AND TABLES

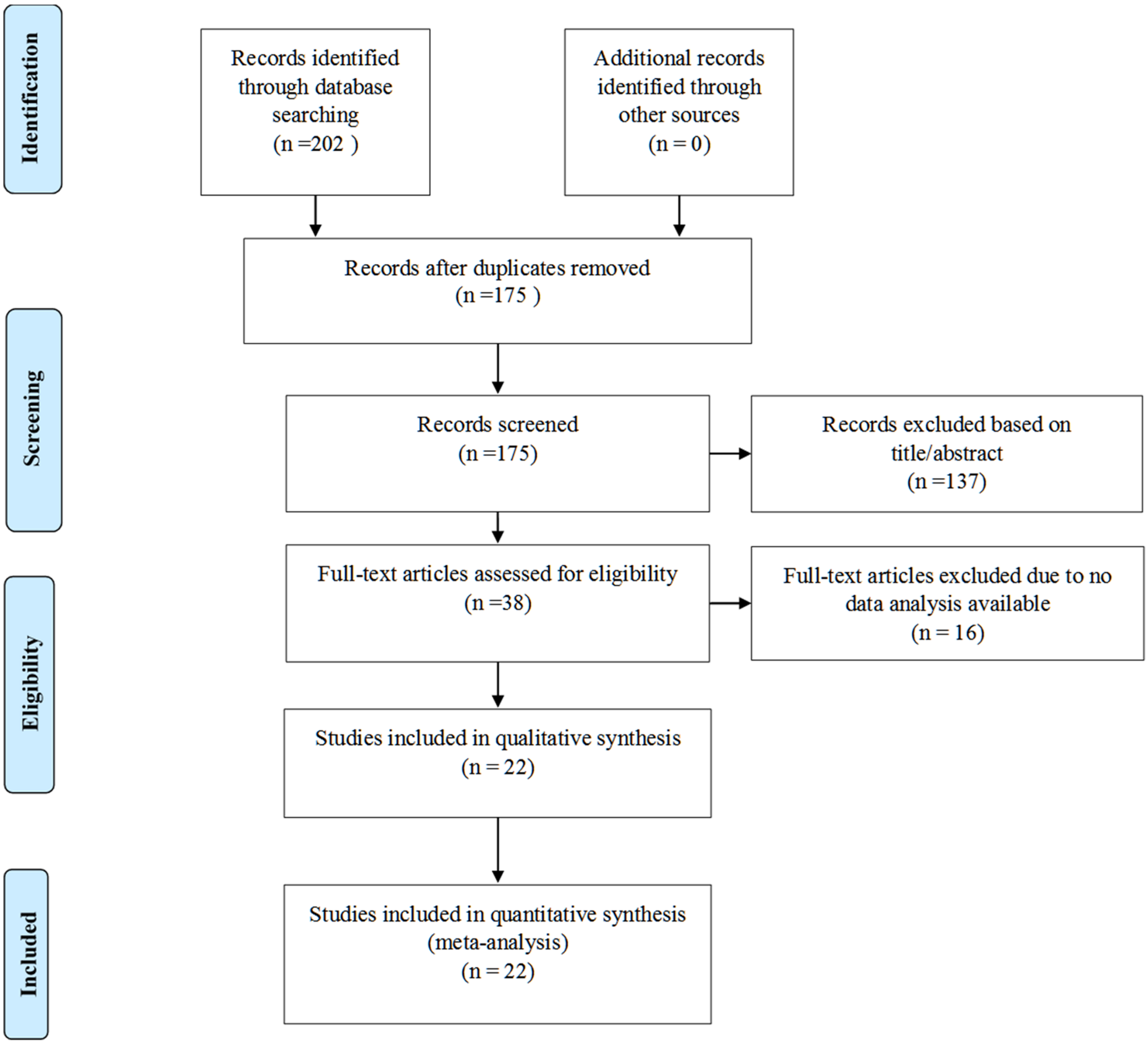

Supplementary Figure 1: PRISMA Flow chart.

Supplementary Table 1: Quality of the studies assessed with the Newcastle Ottawa Scale.

See Supplementary File 1

Supplementary Table 2a: Descriptive characteristics of the studies included.

See Supplementary File 1

Supplementary Table 2b: Data synthesis from TCGA datasets.

See Supplementary File 1

Supplementary Table 3: Type and number of adjustments in each study.

See Supplementary File 1
