# Supplementary material for: Oncofetal gene SALL4 and prognosis in cancer: A systematic review with meta-analysis
Source: Oncotarget. 2017 Feb 1;8(14):22968–79. doi: 10.18632/oncotarget.14952 (PMC5410278; doi:10.18632/oncotarget.14952)
Supplement: Supplementary file 2 [file oncotarget-08-22968-s002.docx]

**Supplementary Table 1. Quality of the studies assessed with the Newcastle Ottawa Scale.**

| **First author, publication year** | **Representativeness of the exposed cohort** | **Selection of the unexposed cohort** | **Ascertainment of exposure†** | **Outcome of interest not present at start of study††** | **Control for important factor or additional factor†††** | **Assessment of outcome** | **Follow-up long enough for outcomes to occur††††** | **Adequacy of follow up of cohorts** | **Total quality scores** |
| --- | --- | --- | --- | --- | --- | --- | --- | --- | --- |
| **Deng G et al. 2015** | * | * | * | - | * | * | - | * | **6** |
| **Han SX et al. 2014** | * | * | * | - | - | * | - | * | **5** |
| **He J et al. 2016** | * | * | * | * | - | * | * | * | **7** |
| **He L. et al. 2012** | * | * | * | - | ** | * | * | * | **8** |
| **Junk YK et al. 2016** | * | * | * | * | * | * | - | - | **6** |
| **Kilic et al. 2016** | * | * | * | * | - | * | - | * | **6** |
| **Li A et al. 2013** | * | * | * | - | - | * | - | * | **5** |
| **Liu L et al. 2014** | * | * | * | * | - | * | - | * | **6** |
| **Liu L et al. 2015** | * | * | * | - | * | * | - | * | **6** |
| **Ma J et al. 2013** | * | * | * | - | - | * | - | * | **5** |
| **Oikawa et al. 2013** | * | * | * | * | - | * | - | - | **5** |
| **Osada M et al. 2014** | * | * | * | - | * | * | - | - | **5** |
| **Park H et al. 2015** | * | * | * | * | ** | * | * | * | **9** |
| **Shibahara J et al. 2014** | * | * | * | * | ** | * | * | * | **9** |
| **Tanaka Y et al. 2015** | * | * | * | * | * | * | * | * | **8** |
| **Wang F et al. 2013** | * | * | * | - | - | * | * | * | **6** |
| **Yang M et al. 2016** | * | * | * | - | ** | * | * | * | **8** |
| **Yin F. et al. 2016** | * | * | * | * | * | * | - | - | **6** |
| **Yong KJ et al. (Hong Kong & Singapore Cohorts), 2014** | * | * | * | * | ** | * | * | * | **9** |
| **Zeng SS et al. 2013** | * | * | * | - | * | * | - | * | **5** |
| **Zhang L et al. 2015** | * | * | * | - | - | * | - | * | **5** |
| **Zhou S et al. 2016** | * | * | * | * | * | * | * | * | **8** |

A study could be awarded a maximum of one star for each item except for the item Control for important factor or additional factor. The definition/explanation of each
column of the Newcastle-Ottawa Scale is available at http://www.ohri.ca/programs/clinical_epidemiology/oxford.htm.
†For this index, one star was given if in Method section the SALL4 assessment was calculated with genetic analysis, whole section-IHC, TMA-IHC with at a least 2 cores
per case or with an adjunctive method or technique as control.
††Being outcome of interest mortality, we took as outcome of interest for assessment of quality if the disease-specific survival or the recurrence rate was assessed.
†††A maximum of 2 stars could be awarded for this item. Studies that controlled their survival analyses for at least two confounders received one star, whereas studies that
controlled genetic status of other gene/genes, an additional star.
††††A cohort study with a mean/median follow-up time ≥ 5 y (60 months) takes one star.

**Supplementary Table 2a. Descriptive characteristics of the studies included.**

| **Author (country), year** | **Type of cancer** | **SALL4 assessment** | **Other genes investigated** | **No. of**  **participants SALL4+** | **Mean age (SD) SALL4+ sample** | **Females (%)** | **TNM stage**  **(%)** | **Tumor grading**  **(%)** | **Nodal metastasis (%)** | **Vascular invasion (%)** | **No. of participants SALL4-** | **Mean age (SD) SALL4- sample** | **Females (%)** | **TNM stage**  **(%)** | **Tumor grading**  **(%)** | **Node metastasis (%)** | **Vascular Invasion (%)** | **Follow-up (months)** | **NOS score** |
| --- | --- | --- | --- | --- | --- | --- | --- | --- | --- | --- | --- | --- | --- | --- | --- | --- | --- | --- | --- |
| Deng G. et al. (China), 2015 | IC | IHC, WB | Ki67, CA19.9, AFP, GGT, TP53 | 102 | 53.52 ± 11.23 | 44 | T1-2: 57, T3-4: 43 | 0:7, 1:75, 2:18 | 25 | 61 | 73 | 56.61 ± 13.26 | 51 | T1-2: 60, T3-4: 40 | 0:8, 1:59, 2:33 | 12 | 8 | 13 | 6 |
| Han SX et al. (China), 2014 | HCC | IHC, ELISA | No | 18 | 50.83 ± 10.33 | 6 | I-II:61, III-IV:39 | 0:17, 1:72, 2:11 | 11 | 22 | 20 | 54.31 ± 14.19 | 10 | I-II:70, III-IV:30 | 0:0, 1:70, 2:30 | 20 | 40 | 40 | 5 |
| He J. et al. (China), 2016 | Esophagous Squamous cells Carcinoma | IHC, RT-PCR, WB | No | 102 | <60:27, >60:75 | 32 | I-IIa:66, Iib-III-IV:36 | 0:23, 1:58, 2:21 | 35 | NA | 31 | <60:12, >60:19 | 14 | I-IIa:26, Iib-III-IV:5 | 0:10, 1:20, 2:1 | 3 | NA | 60 | 7 |
| He L. et al. (China), 2012 | OMGCT | MicroArray, RT-PCR, IHC | KPNA2 | 28 | NA | 100 | NA | NA | NA | NA | 62 | NA | 100 | NA | NA |  | NA | 88 | 8 |
| Jung YK et al. (South Korea), 2016 | HCC | IHC | No | 50 | NA | 66 | I-II:74; III-IV:26 | 0:0, 1:14, 2:74, 3:12 |  | 24 | 163 | NA | 10 | I-II:78;  III-IV:22 | 0:8, 1:42, 2:39, 3:11 | NA | 33 | 53 | 6 |
| Kilic et al. (Germany), 2016 | Esophagous Squamous cells Carcinoma | IHC | No | 14 | NA | NA | NA | 0:0, 1:71, 2:29 | 1 | NA | 138 | NA | NA | NA | 0:2, 1:70, 2:28 | 2 | NA | 24 | 6 |
| Li A et al. (USA), 2013 | Endometrial Cancer | RT-PCR | No | 53 | <50:7.5%, >50:92.5% | 100 | I:66,II: , III: 21,IV:2 (FIGO) | 0:53, 1:26, 2:21 | 9 | 21 | 60 | <50:20%, >50:80% | 100 | I: 77,II: 3, III: 17,IV:3 (FIGO) | 0:48, 1:35, 2:12, Clear Cell:5 | 10 | 3 | 10 | 5 |
| Liu L et al. (USA), 2014 | HCC | IHC (TMA) | No | 3 | 55.6±3.5 | 67 | NA | 0:0, 1:33, 2:67 | NA | NA | 233 | NA | 30 | NA | 0:36, 1:35, 2:29 | NA | NA | 40 | 6 |
| Liu L et al. (China), 2015 | Endometrial Cancer | RT-PCR | N-cadherin, E-cadherin, ABCB1, c-Myc, | 54 | <50:22, >50:34 | 56,00 | I-II:30, III-IV:26 (FIGO) | 0:29, 1:20, 2:7 | 22 | NA | 24 | <50:11, >50:13 | 26 | I-II:22, III-IV:2 (FIGO) | 0:9, 1:9, 2:6 | 4 | NA | 90 | 6 |
| Ma J et al. (China), 2013 | AML | Methylation Status | No | 15 | 66 (20–83) | 38 | M1:40, M2:40, M3:0, M4:13, M5:7, M6:0 | NA | NA | NA | 42 | 43 (15–86) | 69 | M1:9, M2:25, M3:33, M4:26, M5:6, M6:1 | NA | NA | NA | 24 | 5 |
| Oikawa et al. (Japan), 2013 | HCC | MicroArray | No | 110 | NA | NA | NA | NA | NA | NA | 29 | NA | NA | NA | NA | NA | NA | 23 | 5 |
| Osada M et al. (Japan), 2014 | Gastric Adenocarcinoma | IHC | AFP, HepPar1, Glypican 3, PLUNC | 21 | NA | 19 | pT1-pT2=43 pT3-pT4=57 | NA | NA | NA | 24 | NA | 38 | pT1-pT2=25 pT3-pT4=75 | NA | NA | NA | NA | 5 |
| Park H et al. (Korea), 2015 | HCC | IHC (TMA) | K19, EpCam | 39 | <60:51%, >60:49% | 79 | pT1/pT2: 82, pT3a/pT3b/pT4: 18 | I/II:31, III/IV:69 | NA | 44 | 151 | <60:54, >60:46 | 79 | pT1/pT2: 81, pT3a/pT3b/pT4: 19 | I/II:24, III/IV:76 | NA | 36 | 156 | 9 |
| Shibahara J et al. (Japan), 2014 | HCC | IHC | EpCam, CK19 | 47 | 59.2±13.1 | 45 | NA | 0:2, 1:40, 2:58 | NA | 72 | 290 | 52.2 ± 38.7 | 18 | NA | 0:16, 1:66, 2:18 | NA | 54 | 90 | 9 |
| Tanaka Y et al. (Japan), 2015 | cHCC-CC | IHC | AFP, Glypican 3, EpCam, NCAM, OV-6, OCT-4, NANOG | 8 | 62.0 ± 10.3 | 50 | NA | NA | 0 | 50 | 82 | 62.0 ± 11.3 | 18 | NA | NA | 13 | 55 | 120 | 8 |
| Wang F et al. (China), 2013 | MDS, MDS-AML | ICH | No | 23 | NA | NA | NA | NA |  |  | 32 |  |  |  |  |  |  | 24 |  |
| Yang M et al. (China), 2016 | OC | IHC, RT-PCR |  | 53 | <50:60, >50:40 | 100 | FIGO I-II:17; III-IV:83 | Low: 17; High: 83 | 33 |  | 38 | <50:25, >50:13 | 100 | FIGO I-II:58; III-IV:42 | Low: 50; High: 50 | 10 |  | 60 | 8 |
| Yin F. et al. (China), 2016 | HCC | IHC | AFP (serum) | 58 | <50:36, >50:64 | 17 | I-II:79, III-IV:21 | 0:7, 1:39, 2:54 | NA | 21 | 68 | <50:35, >50:65 | 17 | I-II:88, III-IV:12 | 0:13, 1:44, 2:43 | NA | 11 | 23 | 6 |
| Yong KJ et al. (Hong Kong), 2014 | HCC | IHC | No | 228 | <55:46%, >55:54% | 50 | I-II:21, II-IV:79 | 0:6, 1-2:94 | NA | 28 | 114 | <55:39, >55:61 | 17 | I-II:49, III-IV:51 | 0:21, 1-2:79 | NA | 43 | 140 | 9 |
| Yong KJ et al. (Singapore), 2014 | HCC | IHC | No | 45 | <56:49%, >56:51% | 20 | I-II:80, III-IV:20 | NA | NA | 9 | 34 | <56:47, >56:53 | 18 | I-II:82, III-IV:18 | NA | NA | 6 | 250 | 9 |
| Zeng SS et al. (Japan), 2013 | HCC | IHC | EpCam, CK19 | 43 | 60.8 ± 5.4 | 37 | I-II:9, III:77, IV:14 (Edmonton-steiner) | NA | NA | NA | 101 | 64.6 ± 10 | 18 | I-II:18, III:67, IV:15 | NA | NA | NA | 58 | 5 |
| Zhang L et al. (China), 2015 | Glioma | IHC, RT-PCR | No | 39 | <50:56%, >50:44% | 38 | NA | NA | NA | NA | 15 | <50:60, >50:40 | 67 | NA | NA | NA | NA | 120 | 5 |
| Zhou S et al. (USA), 2016 | Hepatoblastoma | IHC | No | 52 | NA | 40 | NA | NA | 16 | 16 | 27 | NA | 33 | NA | NA | 9 | 9 | 120 | 8 |
| **Total** | **11: HCC; 2: endometrial; 2: esophageal cancer;**  **1: glioma; 1: IC; 1:OMGCT; 1: OC; 1: hepatoblastoma; 1: gastric adenocarcinoma; 1: AML** | **18: IHC; 4: RT-PCR; 1: methilation status; 1: microarray** | **-** | **1,277** | **58.3±16.1** | **50** | **I-II: 52%; III-IV: 48%** | **0: 25%; 1: 44%; 2: 31%** | **14.8%** | **36.1%** | **1,918** | **56.1±**  **16.8** | **47** | **I-II: 60%; III-IV: 40%** | **0: 27%; 1: 48%; 2: 25%** | **13.7%** | **34.1%** | **Median=55.7 (range: 10-250) months** |  |

**Abbreviations:** SD: standard deviation, HCC: hepatocellular carcinoma, SALL4: Spalt-Like Transcription Factor 4, IHC: immunohistochemistry, RT-PCR: real time-polymerase chain reaction, AML: acute myeloid leukemia, NOS: New Castle – Ottawa quality score, TNM: Tumor Staging system, FIGO: International Federation of Gynecology and Obstetrics staging score, CK19: cytokeratin 19, CA19.9: carbohydrate antigen 19.9, AFP: alfa-fetoprotein, GGT: gamma-glutaril transferase, EpCam: Epithelial cell adhesion molecule, OCT4: octamer-binding transcription factor 4,OV-6: oval cells marker, PLUNC: BPI fold containing family A member 1, KPNA2: Karyopherin Subunit Alpha 2, ABCB1: ATP binding cassette subfamily B member 1, cHCC-CC: combined hepatocellular carcinoma and cholangiocarcinoma, IC: Intrahepatic Cholangiocarcinoma, OMGCT: ovarian malignant germ cell tumor, pT: pathologic Tumor stage, NA: not available, ELISA: enzyme-linked immunosorbent assay, WB: western blotting, TMA: tissue micro array.

**Supplementary table 2b: data synthesis from TCGA datasets:**

| **TCGA dataset** | **No. of participants SALL4+** | **Mean age (SD) SALL4+ sample** | **Females (%) SALL4+** | **TNM stage (%) SALL4+** | **Tumor grading (%) SALL4+** | **No. of participants SALL4-** | **Mean age (SD) SALL4- sample** | **Females (%) SALL4-** | **TNM stage (%) SALL4-** | **Tumor grading (%) SALL4-** | **Follow-up (months)** |
| --- | --- | --- | --- | --- | --- | --- | --- | --- | --- | --- | --- |
| BLCA | 31,00 | 68,71 | 26 | AJCC;I-II:33;III:30,IV:37 | Low:0;High:100 | 373,00 | 67,96 | 26 | AJCC;I-II:32;III:35,IV:33 | Low:5;High:95 | 30,28 |
| BRCA | 76,00 | 56,74 | 100 | AJCC; I:1;II:58;III:31 | NA | 1013,00 | 58,59 | 99 | AJCC;I:17;II=57;III:22;IV:2 | NA | 22,85 |
| CESC | 39,00 | 48,74 | 100 | I:61;II:26;III:13 | I:8;II:42;;III:42;X:8 | 257,00 | 47,86 | 100 | I=53;II=23;III=16;IV=8 | I:6;II=47;III=39;X=8 | 11,37 |
| COADREAD | 39,00 | 66,31 | 41 | AJCC;I:9;II:34;III:48;IV:9 | NA | 334,00 | 64,32 | 45 | AJCC;I:17;II:38;III:30;IV:15 | NA | 19,08 |
| ESCA | 24,00 | 62,04 | 13 | AJCC;I:10;II:35;III:55 | II:25;III:46;X:29 | 147,00 | 61,61 | 14 | AJCC;I:11;II:52;III:30;IV:7 | I:12;II:45;III:23;X:20 | 7,00 |
| GBM | 17,00 | 66,53 | 24 | - | - | 138,00 | 59,05 | 36 | - | - | 18,00 |
| HNSC | 31,00 | 61,45 | 16 | II:19;III:10;IV:71 | I:6;II:52;III:32;X:10 | 472,00 | 60,86 | 27 | I:4;II:19;III:22;IV:55 | I:12;II:59;III:24;IV:2;X:3 | 26,80 |
| KIRC | 20,00 | 59,25 | 35 | AJCC;I:25;II:5;III:30;IV:40 | II:20;III:40;IV:40 | 504,00 | 60,63 | 35 | AJCC;I:51;II:11;III:23;IV:15 | I:3;II:44;III:39;IV:14 | 42,63 |
| LIHC | 33,00 | 53,39 | 52 | AJCC;I:44;II:22;III:34 | I:3;II:30;III:64;IV:3 | 337,00 | 60,03 | 31 | AJCC;I:50;II:25;III:24;IV:1 | I:16;II:50;III:30;IV:4 | 60,00 |
| LUAD | 33,00 | 66,45 | 55 | AJCC;I:58;II:21;III:15;IV:6 | NA | 472,00 | 65,28 | 54 | AJCC;I:54;II:24;III:17;IV:5 | NA | 36,89 |
| LUSC | 49,00 | 66,63 | 20 | AJCC;I:47;II:41;III:10;IV:2 | NA | 439,00 | 67,30 | 27 | AJCC;I:50;II:31;III:18;IV:1 | NA | 13,30 |
| OV | 22,00 | 62,73 | 100 | II:18;III:64;IV:18 | II:9;III:82;X:9 | 280,00 | 58,78 | 100 | I:1;II:6;III:81;IV:12 | II:11;III:87;IV:1;X:1 | 33,29 |
| SARC | 12,00 | 55,92 | 58 | NA | NA | 246,00 | 60,91 | 54 | NA | NA | 152,00 |
| STAD | 111,00 | 67,13 | 33 | AJCC;I:12;II:26;III:55;IV:7 | I:4;II:48;III:48 | 286,00 | 65,11 | 37 | AJCC;I:14;II:34;III:40;IV:12 | I:3;II:31;III:66 | 55,00 |
| THCA | 22,00 | 56,09 | 55 | AJCC;I:41;II:18;III:23;IV:18 | NA | 478,00 | 46,81 | 74 | AJCC;I:58;II:10;III:22;IV:10 | NA | 39,75 |
| UCEC | 13,00 | 67,00 | 100 | I:54;II:8;III:23;IV:15 | I:8;III:84;IV:8 | 158,00 | 65,47 | 100 | I:56;II:14;III:25;IV:5 | I:8;II:13;III:77;IV:2 | 100,00 |
| SKCM | 34,00 | 56,41 | 24 | AJCC;I:10;II:28;III:52;IV:10 | NA | 324,00 | 56,23 | 39 | AJCC;I:26;II:23;III:45;IV:6 | NA | 23,33 |

**Supplementary Table 3. Type and number of adjustments in each study.**

| **Study (country)** | **Type of adjustments** | **Number of adjustments** |
| --- | --- | --- |
| A Li et al. (USA), 2013 | - | - |
| Deng G. et al. (China), 2015 | - | - |
| Han SX et al. (China), 2014 | - | - |
| He J. et al. (China), 2016 | - | - |
| He L. et al. (China), 2012 | KPNA2, FIGO Stage, Cytoreductive surgery, Response to initial chemotherapy | 4 |
| J. Ma et al. (China), 2013 | - | - |
| Junk YK et al. (South Korea), 2016 | - | - |
| Kilic et al. (Germany), 2016 | - | - |
| Liu et al. (USA), 2014 | - | - |
| Liu L et al. (China), 2015 | - | - |
| M. Osada et al. (Japan), 2014 | - | - |
| Oikawa et al. (Japan), 2013 | - | - |
| Park H et al. (Korea), 2015 | SALL4 positivity, Multiplicity of tumor, High pT stage | 3 |
| S.S. Zeng et al. (Japan), 2013 | - | - |
| Shibahara J et al. (Japan), 2014 | Age, Sex, HBV, HCV, Child-Pugh, AFP, Tumor size, Histologic grade, Vascular invasion, IM, Background liver, SALL4, CK19, EpCAM | 14 |
| Tanaka Y et al. (Japan), 2015 | - | - |
| Yang M et al. (China), 2016 | Clinical stage, histological differentiation, BCLC stage, AFP, Tumor size, Vascular invasive | 6 |
| Yin F. et al. (China), 2016 | Clinical stage, histological differentiation, BCLC stage, AFP, Tumor size, Vascular invasive | 6 |
| Yong KJ et al. (Hong Kong), 2014 | Serum AFP, BCLC stage | 5 |
| Yong KJ et al. (Singapore), 2014 | Serum AFP, BCLC stage | 2 |
| Zhang L et al. (China), 2015 | Pathology grade, Extent of resection | 2 |
| Zhou S et al. (USA), 2016 | Gender, Age, Tumour size, Lymphovascular invasion, Metastasis, Metastasis, Viable tumour after chemo, Serum AFP, Transplant, Prematurity | 10 |
| BLCA (TCGA) | Age (*), Gender, Grade (^), History of Other Malignancy, Race | 1 |
| BRCA (TCGA) | Age (*), History of Other Malignancy (^) | 1 |
| CESC (TCGA) | Age, Clinical stage (*^) | 1 |
| COADREAD (TCGA) | Age (*), Gender (^), History of Other Malignancy | 1 |
| ESCA (TCGA) | Age, Gender, Race (^), Tumor Status (*^), Weight | 1,2 |
| GBM (TCGA) | Age (*^), Gender | 1 |
| HNSC (TCGA) | Age, Clinical stage (^), Gender (*), Grade, History of Other Malignancy, Race (*) | 2,1 |
| KIRC (TCGA) | Age (*), Gender, Grade (*^), Neoadjuvant Therapy Type Administered Prior To Resection (*), History of Other Malignancy, Race | 3,1 |
| LIHC (TCGA) | Age (*), Gender, Grade, History of Other Malignancy, Race (^) | 1 |
| LUAD (TCGA) | Gender, History of Other Malignancy (*^) | 1 |
| LUSC (TCGA) | Age (*), Grade, History of Other Malignancy(^) | 1 |
| OV (TCGA) | Age (*), Clinical stage (^), Grade, Race | 1 |
| SARC (TCGA) | Age, Gender (*), History of Other Malignancy (^), Race | 1 |
| SKCM (TCGA) | Age (*^), Gender, Neoadjuvant Therapy Type Administered Prior To Resection, History of Other Malignancy, Radiation Treatment (^) | 1,2 |
| STAD (TCGA) | Age, Gender (^), Grade (*^) | 1,2 |
| THCA (TCGA) | Age (*^), Gender, History of Other Malignancy | 1 |
| UCEC (TCGA) | Age (*^), Clinical Stage (*^), Grade | 2 |
